# Polarization‐Independent Single‐Band Guided‐Mode Resonance InGaAs Photodetector Array for Snapshot Multispectral Sensing

**DOI:** 10.1002/nap2.70297

**Published:** 2026-09-21

**Authors:** Junho Jang, Hosang Hwang, SangHyeon Kim

**Affiliations:** ^1^ School of Electrical Engineering Korea Advanced Institute of Science and Technology (KAIST) Daejeon Republic of Korea

**Keywords:** 2D grating, guided‐mode resonance, InGaAs photodetector, multispectral sensing, short‐wave infrared

## Abstract

Multispectral sensing in the short‐wave infrared (SWIR) range is highly desirable for a wide range of applications, from industrial inspection to satellite remote sensing. Here, we report a single‐band guided‐mode resonance (GMR) InGaAs photodetector (PD) array that enables single‐snapshot multispectral sensing through direct readout from each pixel. The device consists of an InGaAs PD with an ultrathin absorption layer bonded onto a Si 2D grating. Although a 2D crossed‐grating provides polarization independence, it inherently supports many more optical modes than a 1D grating. To realize a single‐band response, we introduce design strategies that suppress higher‐order diffraction using a thin grating and eliminate undesired modes by positioning their diffraction angles near the critical angle. The device exhibited a single‐band spectral response over a broad wavelength range of 1100–1700 nm, peak responsivities of up to 0.451 A/W, off‐peak responsivities below 0.03 A/W, and complete polarization independence. Moreover, a multispectral pixel array can be fabricated using a single patterning step by varying the grating period. We verified the feasibility of snapshot multispectral sensing through area‐scan imaging measurements under various input conditions. These results, together with the fabrication simplicity, establish the proposed device as a promising platform for compact, real‐time SWIR multispectral sensors.

## Graphical abstract

Here, we report a single‐band guided‐mode resonance (GMR) InGaAs photodetector (PD) array. The device consists of an InGaAs PD with an ultrathin absorption layer bonded onto a Si 2D grating. Although a 2D crossed‐grating inherently supports many more optical modes than a 1D grating, we realized a single‐band response by introducing novel design strategies.

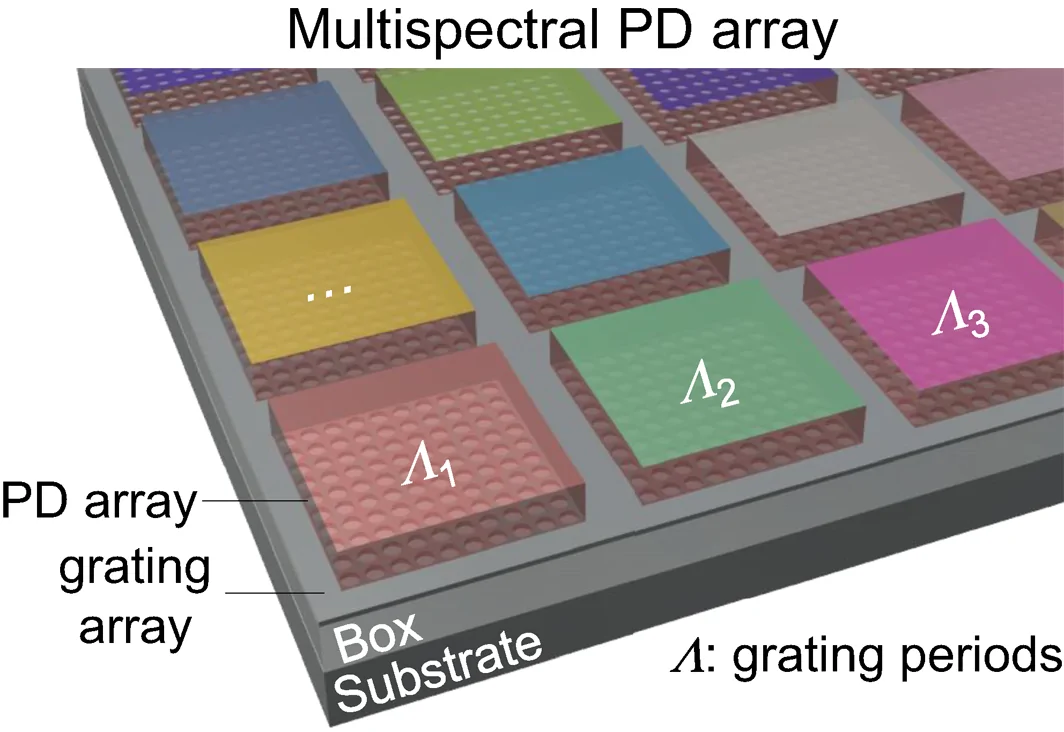

## 1 Introduction

Conventional photodetectors (PDs) typically detect light across a wide spectral range, mainly within the visible region. In recent years, however, the growing need for advanced optical sensing has driven interest in multispectral sensing, which enables detection at multiple distinct wavelength bands extending beyond the visible spectrum [1, 2, 3, 4]. Particularly, multispectral sensing in the short‐wave infrared (SWIR) region is important for applications including biomedical diagnostics, remote sensing, environmental monitoring, optical communications, and industrial inspection. This importance arises from the characteristic absorption features of hydrogen‐bonded materials in the SWIR range, as well as the relatively high transmission of SWIR light through various media [5, 6, 7, 8]. However, conventional multispectral sensing systems rely on bulky optical components such as filter wheels, beam splitters, and multiple cameras, and often require lengthy scanning processes, which significantly limit sensing speed. As an alternative, single‐snapshot multispectral sensing can be achieved using an array of pixels with single‐band spectral responses, where the spectral intensity is obtained by direct readout from each pixel [9, 10, 11]. To realize single‐band pixels for multispectral pixel arrays, numerous devices employing various materials and structures have been reported, including resonance cavities [12, 13, 14, 15], perovskites [16, 17, 18, 19, 20, 21, 22], organic semiconductors [14, 23, 24], quantum dots [15, 25, 26], and nanowires [27, 28]. However, most of these approaches require variation in cavity thickness or material composition for each spectral band, which makes the fabrication of multispectral pixel arrays difficult.

Because implementing single‐band multispectral pixel arrays is challenging, spectral sensing devices based on computational reconstruction using pixel arrays with arbitrary spectral responses have recently attracted considerable attention [29, 30, 31, 32, 33, 34, 35, 36]. In such systems, each pixel is not required to exhibit an ideal single‐band spectral response; instead, the incident spectrum is reconstructed from the collective outputs of multiple pixels. The reconstruction accuracy can be improved by increasing the number of pixels. Accordingly, spectral sensing systems employing large‐scale pixel arrays with up to 150 pixels have been demonstrated [30]. These approaches generally require precisely controlled input conditions and additional computational processing for spectral reconstruction compared to direct readout, which can limit their suitability for real‐time sensing. Therefore, such devices have been studied primarily as reconstructive spectrometers for static analysis.

To enable multispectral pixel arrays, guided‐mode resonance (GMR) presents a promising approach. GMR is an optical resonance phenomenon observed in waveguide–grating structures [37]. The structure of a multispectral pixel array based on GMR PDs is illustrated in Figure 1a. By exploiting GMR, pixels with narrowband spectral responses can be fabricated through tailored grating designs. Moreover, by splitting the grating pattern, an array in which each pixel has a different detection wavelength can be fabricated using only a single patterning and etching step. If each GMR PD pixel exhibits a single‐band response over the target wavelength range (Figure 1b), snapshot multispectral sensing becomes possible through direct readout from each pixel (Figure 1c). However, previously reported GMR PDs have not demonstrated sufficient wavelength selectivity for spectral sensing [38, 39, 40, 41, 42, 43]. Conventional GMR PDs generally consist of a 1D grating structure formed on top of the PD layer, which acts as a waveguide slab, as shown in Figure 1d. In our previous work, we introduced a GMR PD with a new structure in which an InGaAs PD is bonded onto a 1D Si grating (Figure 1e) [44]. In this structure, the Si grating layer and the PD layer jointly form the slab, enabling high resonance efficiency. Furthermore, the InGaAs absorption layer is designed to be extremely thin to minimize absorption outside the resonance. However, owing to the characteristics of 1D GMR, strong polarization dependence is observed, and TE and TM peaks appear at adjacent wavelengths, which significantly limits the single‐band operating range. Polarization dependence and the limited single‐band operating range remain unresolved challenges for GMR PDs [38, 39, 40, 41, 42, 43, 44] and GMR filters [45, 46, 47]. We previously demonstrated the first reconstructive spectrometer based on an array of GMR PDs with diverse spectral responses [36]. However, snapshot multispectral sensing through direct readout of a single‐band GMR pixel array has not yet been realized. When a 2D crossed grating is introduced instead of a 1D grating in a GMR device, polarization‐independent operation can be achieved because the grating structure is symmetric along two orthogonal axes. In 2024, N. C. Mansfield et al. reported an InAsSb photodetector in which absorption enhancement was achieved through the overlap of multiple optical modes supported by a top GaSb 2D grating [42]. However, a 2D grating inherently supports many more optical modes than a 1D grating [42, 43, 48], making it extremely difficult to realize narrow single‐band characteristics over a broad wavelength range.

**FIGURE 1:**
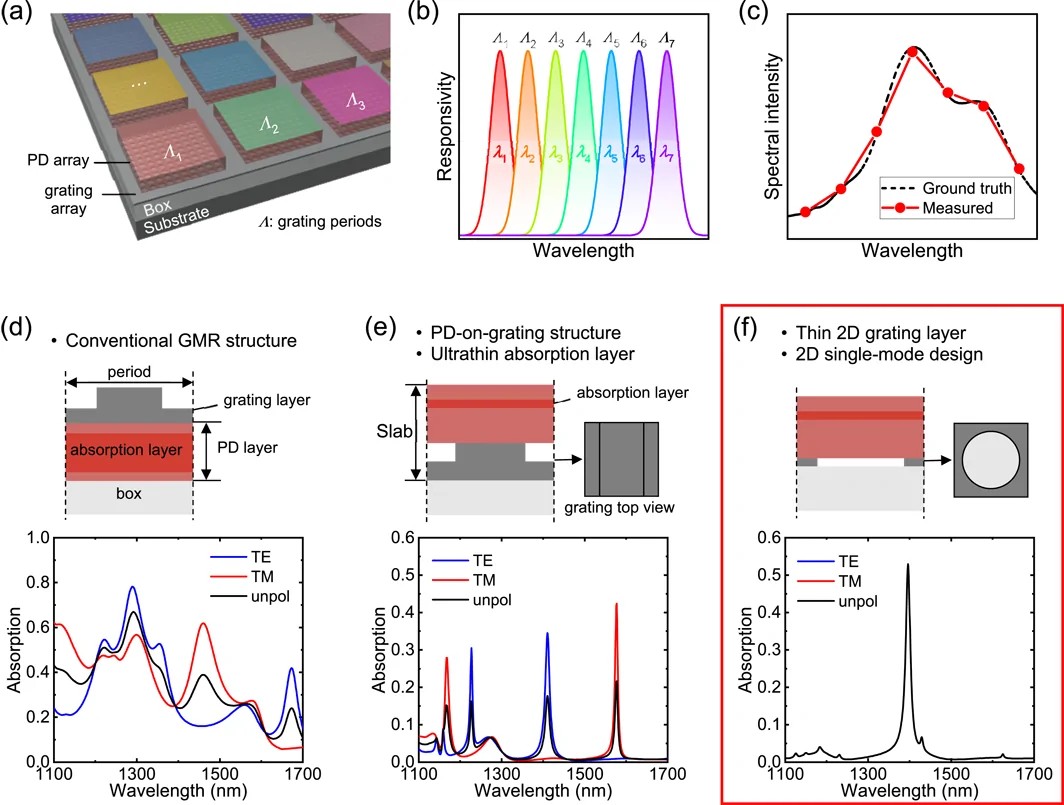
(a) Schematic of the GMR PD array. (b) Ideal spectral responses of a single‐band GMR PD array according to the grating period. (c) Obtained spectral intensity through direct readout from the multispectral GMR PD array. Schematic and absorption spectrum of (d) conventional GMR PDs with typical structure, (e) previously reported 1D GMR PD with improved narrowband detection [44], and (f) proposed polarization‐independent single‐band 2D GMR PD.

In this study, we propose a polarization‐independent, single‐band GMR photodetector based on a 2D crossed grating that enables snapshot multispectral sensing (Figure 1f). Although 2D GMR structures inherently support multiple optical modes, we achieved a single‐band response over a broad wavelength range from 1100 to 1700 nm by employing a new design strategy: suppressing higher‐order modes using an ultrathin grating and placing the diffraction angles of undesired modes close to the critical angle. A PD array with varied grating periods was fabricated, and its performance was experimentally validated, demonstrating peak responsivities of up to 0.451 A/W, off‐peak responsivities below 0.03 A/W, and complete polarization independence. In addition, through area‐scan imaging measurements under light with various spectra and polarization states, we demonstrated the capability of snapshot multispectral sensing using the proposed GMR PD array. The superior single‐band detection performance, together with the fabrication simplicity that allows multispectral pixel arrays to be fabricated using a single patterning and etching step, makes this device a promising candidate for compact, real‐time multispectral sensors.

## 2 Device Concept and Design Strategy

The structure and resonance mechanism of the proposed GMR PD are illustrated in Figure 2a. The device consists of an InGaAs PD bonded onto a Si grating. When the incident light reaches the Si grating, it is diffracted into multiple directions by the periodic structure. The diffraction of light by the 2D crossed Si grating is illustrated in Figure 2b. For normal incidence, the diffraction angle of the (*p*,*q*) order diffraction is expressed as follows:

(1) $$\theta_{\left(p,q\right)}=\sin^{-1}\left(\frac{\sqrt{p^{2}+q^{2}}\lambda_{0}}{n_{\text{eff}}\Lambda }\right),$$

where *p* and *q* are integers, *λ*_0_ is the wavelength in vacuum, *n*_eff_ is the effective refractive index of the slab, and *Λ* is the grating period. Because the refractive index of Si is similar to that of the PD materials, the PD layer and Si grating layer together form a waveguiding slab. The diffracted light undergoes repeated total internal reflections within the slab, and constructive interference of the internally reflected beams at specific wavelengths leads to resonance and enhanced absorption. This is referred to as the waveguiding condition, and the equation obtained through light‐path analysis is expressed in ref. [37] as follows:

(2) $$2\pi N=2k_{0}n_{\text{eff}}h\;\cos\;\theta_{\left(p,q\right)}+\phi_{1}+\phi_{2},$$

where *k*_0_ is the wavenumber in a vacuum, *N* is an integer representing the GMR order, *h* is the height of the slab, and $\phi_{1}$, $\phi_{2}$ are the phase shifts during the total internal reflection. The phase shift has different values for TE and TM polarizations, which is the origin of the polarization dependence in 1D GMR devices. Nevertheless, in a 2D crossed grating, TE and TM modes are excited with equal strength owing to structural symmetry, resulting in a polarization‐independent spectral response. At the resonance wavelength, absorption in the active layer is enhanced, and the light absorption is effectively concentrated in the absorption layer because all materials in the slab other than InGaAs are transparent over the SWIR range. To minimize off‐resonance absorption, the InGaAs absorption layer was designed to be very thin (18 nm). As the thickness of the absorption layer increases, off‐resonance absorption increases, whereas the peak absorption decreases beyond a certain thickness. A thicker absorption layer causes more light to be absorbed during each round trip within the slab, thereby reducing the number of round trips and consequently weakening the resonance (Supporting Information S1: Figure S1). Figure 2c shows the simulated electric‐field distribution at the resonance wavelength. The simulation exhibits the typical characteristics of first‐order GMR. To maximize absorption, the InGaAs absorption layer should be positioned close to the field maximum at resonance.

**FIGURE 2:**
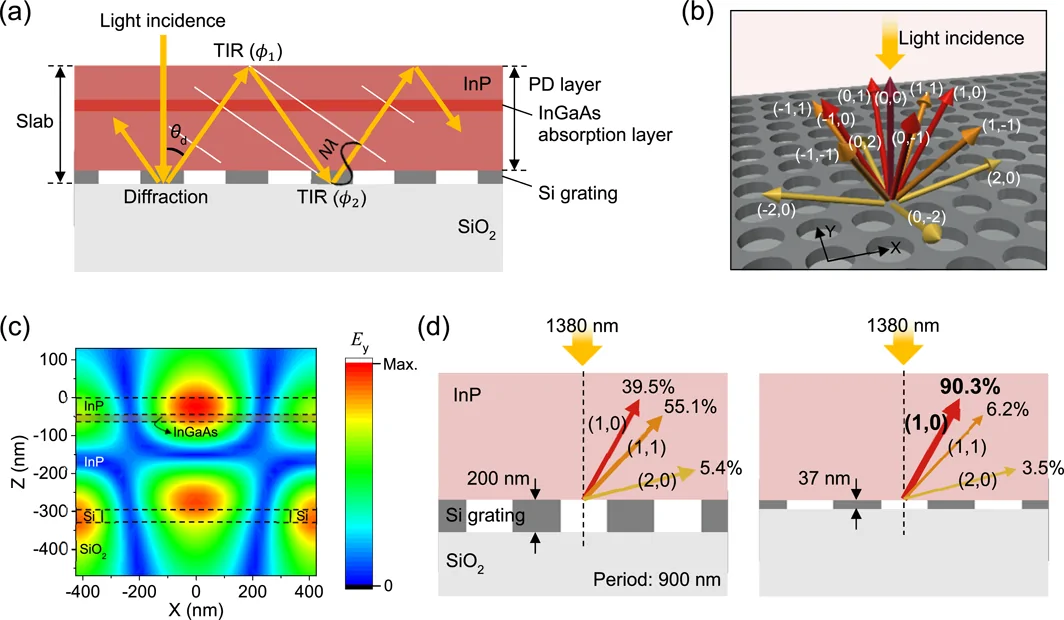
(a) Structure and resonance mechanism of the GMR PD. (b) Schematic describing the light diffraction by a 2D crossed grating. (c) Simulated electric field distribution within the device at resonance. (d) Results of diffraction simulations according to the grating height. Portions of the (1,0), (1,1), and (2,0) diffractions are indicated.

To achieve single‐band operation, only one resonance peak should exist within the operational wavelength range. As a first strategy for single‐band operation, the Si grating thickness was designed to be very thin (37 nm) in order to suppress higher‐order diffractions other than the (1,0). Figure 2d shows the simulated diffraction efficiency as a function of grating height. The simulation assumes normally incident 1380 nm light entering from the upper InP medium onto a grating with a period of 900 nm. When the grating thickness is 200 nm, the diffraction efficiencies are distributed relatively evenly among several orders. However, when the thickness is reduced to 37 nm, diffraction into orders other than (1,0) becomes almost negligible. As the grating thickness decreases, the diffraction efficiency decreases because the periodic structure introduces weaker refractive‐index modulation to the incident light. Higher‐order diffractions are more strongly affected by shallow gratings than lower‐order diffractions, because they rely on higher‐spatial‐frequency Fourier components of the grating profile.

Next, GMR mode analysis was carried out to establish a precise structural design for single‐band operation. Figure 3a plots the GMR modes occurring in the device over the wavelength range of 1100–1700 nm as a function of PD (InP‐InGaAs‐InP multilayer) thickness for a grating period of 850 nm. The wavelength of each mode was calculated from Equations (1) and (2). In the graph, the GMR order is distinguished by line color, whereas the diffraction order is distinguished by line type. In this device, higher‐order diffraction modes, other than the (1,0) modes indicated by thick solid lines, can be suppressed by introducing a thin grating layer. In this design, the (1,0) TE first‐order mode is regarded as the main mode. To realize a single‐band response, it is essential to suppress the TM mode paired with the main mode, namely, the (1,0) TM first‐order mode. Figure 3b plots the diffraction angles within the slab for each mode as a function of PD thickness, with the critical angle at the slab–SiO_2_ interface indicated by a vertical dashed line. The critical angle varies slightly depending on the wavelength because of the wavelength‐dependent refractive indices; an averaged value is indicated. When the diffraction angle approaches the critical angle, optical loss is induced during repeated internal reflections, thereby preventing the formation of a resonant mode. Therefore, we designed the total PD thickness to be 290 nm so that the diffraction angle of the undesired mode becomes close to the critical angle. The simulated spectral absorption as a function of PD thickness is plotted in Figure 3c. The simulation was performed using the rigorous coupled‐wave analysis (RCWA) method, with an absorption monitor placed in the InGaAs active layer. A comparison of Figure 3a–c confirms that when the diffraction angle of a given mode approaches the critical angle, the corresponding absorption peak is suppressed. In contrast, for the main mode, the diffraction angle remains sufficiently larger than the critical angle, and the resonance therefore appears clearly. As a result, only a single absorption peak remains at a PD thickness of 290 nm. This diffraction‐angle engineering is the key strategy for designing the single‐band GMR device proposed in this work.

**FIGURE 3:**
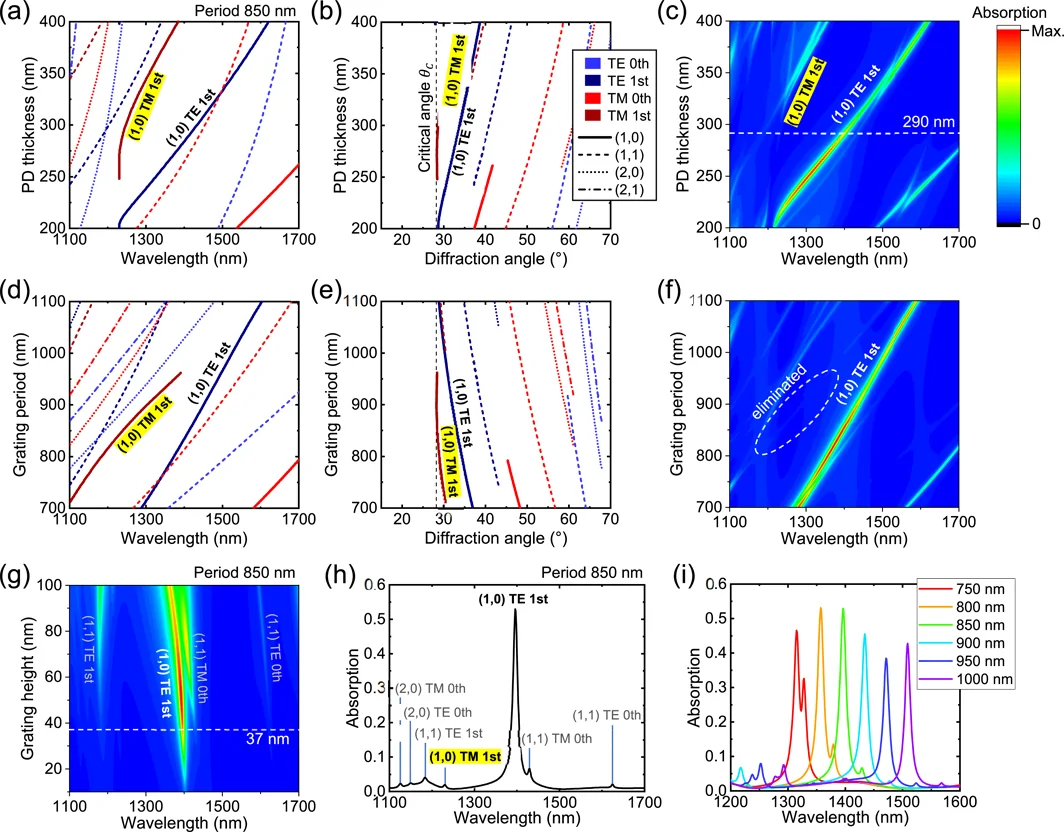
(a) Calculated GMR modes plotted according to the PD (InP‐InGaAs‐InP multilayer) thickness. (b) Diffraction angles within the slab for each mode plotted according to the PD thickness. The averaged critical angle at the slab–SiO_2_ interface is indicated by a vertical dashed line. (c) Spectral absorption simulation results according to the PD thickness. (d) Calculated GMR modes plotted according to the grating period. (e) Diffraction angles for each mode plotted according to the grating period. (f) Spectral absorption simulation results corresponding to (d). (g) Spectral absorption simulation results according to the grating height. (h) Simulated spectral absorption for a grating period of 850 nm, with all existing modes indicated. (i) Absorption simulation results for several grating periods ranging from 750 to 1000 nm.

Figure 3d,e plot the GMR modes and the diffraction angles of each mode, respectively, as a function of grating period. The absorption simulation results corresponding to the mode calculation in Figure 3d is shown in Figure 3f, confirming that the undesired TM mode is completely suppressed over a wide range of grating periods, leaving only a single main mode. The wavelength at which the main mode occurs varies with grating period, indicating that a multispectral pixel array with various detection wavelengths can be realized by varying only the grating period without modifying any other parameters. Figure 3g presents absorption simulation results for varying grating heights. As the grating height is reduced, higher‐order diffraction modes are suppressed because a shallow grating strongly inhibits the generation of higher‐order diffractions, as shown in the diffraction simulations in Figure 2d. The grating height was designed to be 37 nm because an excessively thin grating would also reduce the diffraction efficiency of the main mode. Absorption simulation results according to the duty cycle are presented in Supporting Information S1: Figure S2c. Through optimization based on absorption simulations, we determined the grating structure to be a circular‐hole grating with a duty cycle of 0.8. Figure 3h presents the simulated spectral absorption for a grating period of 850 nm, with all existing modes indicated. A single‐band GMR PD was successfully designed by minimizing higher‐order diffraction through the use of a thin grating layer and suppressing the undesired mode through diffraction‐angle engineering. Figure 3i presents absorption simulation results for several grating periods ranging from 800 to 1050 nm. Depending on the grating period, the peak absorption ranges from approximately 0.4–0.5. This variation is mainly attributed to the Fabry–Pérot cavity effect and can therefore be adjusted within this range by adjusting the box‐layer thickness. Single‐band spectral responses are obtained for all grating periods, confirming the feasibility of implementing a multispectral pixel array through grating‐period variation. The unit‐cell structure and detailed dimensions of the designed GMR PD are provided in Supporting Information S1: Figure S4. Because the active InGaAs layer is only 18 nm thick, intrinsic InP layers were inserted above and below the InGaAs layer to facilitate the formation of a stable *p*–*i*–*n* junction. The band diagram of the InP–InGaAs photodetector structure is shown in Supporting Information S1: Figure S5. Although the InP/InGaAs band offsets confine photogenerated carriers within the InGaAs layer, the strong electric field across the thin intrinsic region facilitates carrier escape through field‐assisted transport and tunneling, enabling efficient conversion of the electron–hole pairs generated in the InGaAs active layer into photocurrent.

## 3 Device Fabrication and Measurement

The epitaxial structures of the InGaAs PD substrate and the overall fabrication process are illustrated in Supporting Information S1: Figures S6 and S7. A 1 μm–thick SiO_2_ layer and a 37 nm–thick amorphous (a‐) Si layer were deposited on a Si substrate, and the grating was formed by photolithographic patterning followed by Si dry etching. The grating substrate contains circular‐hole 2D crossed‐grating pixels with various grating periods and, for comparison, includes 1D grating patterns (duty cycle 0.5) and 2D grating patterns with different shapes, such as square‐hole and diamond gratings. The Si grating substrate and the PD substrate were bonded via plasma‐activated wafer bonding using a 10 nm–thick alumina layer as the bonding material. The PD array was then formed by pixel patterning followed by wet etching processes. Each pixel was fabricated with dimensions of 168 μm × 168 μm, and the size of the optical window was 150 μm × 150 μm. During mesa etching, a portion of the *n*‐InP layer was left unetched to provide the bottom electrical contact. The detailed fabrication process is described in the Experimental Section, and the detailed structure of an individual pixel is presented in Supporting Information S1: Figure S8. Images of the fabricated GMR PD array and Si grating substrate are shown in Figure 4a. A magnified scanning electron microscope (SEM) image of the Si grating is presented in Figure 4b. The circular‐hole grating provides not only the highest performance but also the best fabrication reproducibility. Focused ion beam (FIB) cross‐sectional SEM images of the GMR PD are presented in Figure 4c, demonstrating high‐quality bonding interfaces. After fabrication, current–voltage (*I–V*) measurements were conducted under dark conditions and under 1310 nm laser diode illumination (Figure 4d). The measurements were performed on a pixel with a grating period of 750 nm, for which 1310 nm lies near the peak absorption wavelength. The device exhibited a low dark current of approximately 40 nA/cm^2^ at −0.5 V, which is within the range reported for high‐quality InGaAs/InP photodiodes [49]. The device maintained a nearly constant photoresponse with increasing reverse bias voltage. In Figure 4e, the measured photocurrents at 0 V are plotted as a function of light power. The linear fit on a log–log scale yielded a slope close to unity, indicating a very low defect density at the junction. Because the device is electrically a single‐crystal InGaAs *p*–*i*–*n* diode, it provides superior electrical performance compared with devices based on non‐traditional semiconductor materials [14, 15, 16, 17, 18, 19, 20, 21, 22, 23, 24, 25, 26, 27, 28].

**FIGURE 4:**
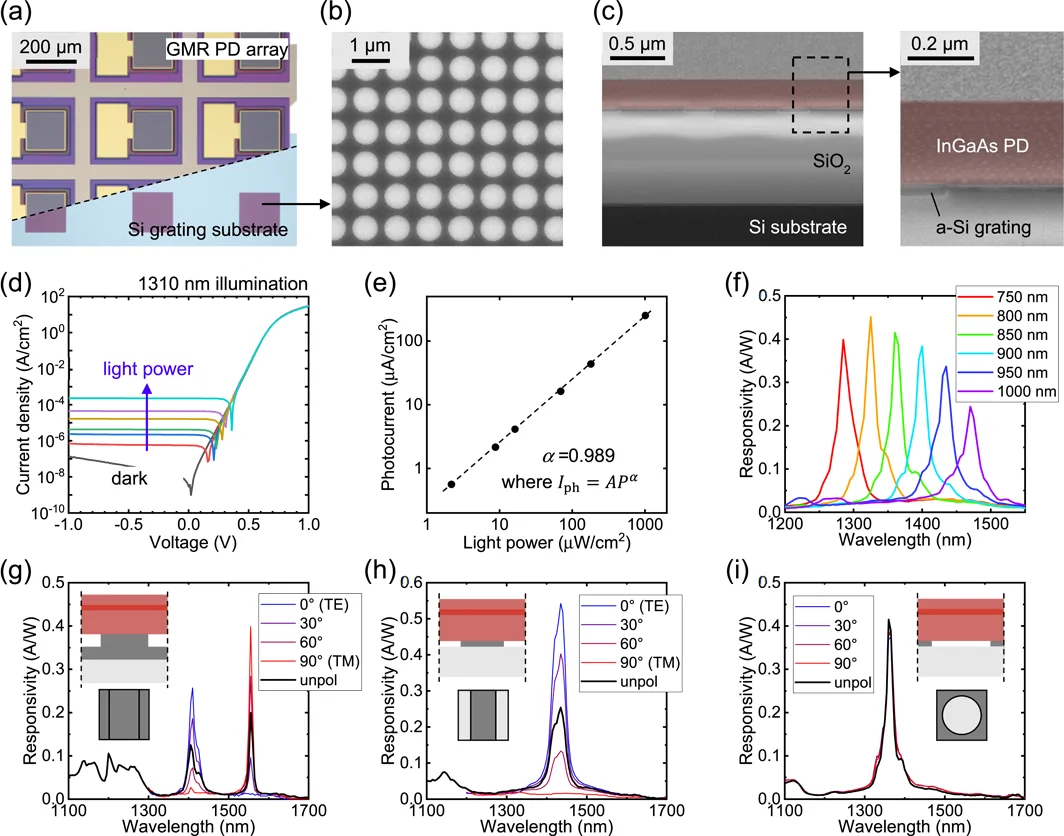
(a) Optical microscope images of fabricated GMR PD array and Si grating array. (b) A magnified scanning electron microscope (SEM) image of the Si grating. (c) Focused ion beam (FIB) cross‐sectional SEM images of the GMR PD. (d) Results of I‐V measurements under dark and 1310 nm laser diode illumination, for a pixel with a grating period of 750 nm. (e) Photocurrent at 0 V as a function of optical power, fitted with a power law: *I*_ph_ = *AP* ^*α*^, where *I*_ph_ is photocurrent, *P* is light intensity, and *A* and *α* are coefficients that depend on the characteristics of a device. (f) Spectral responsivities of pixels with grating periods from 750 to 1000 nm, measured under unpolarized illumination using a monochromator scan. (g)‐(i) Polarization‐dependent spectral responsivities measured while rotating a linear polarizer in 30 increments, for (g) a pixel from the previously reported 1D grating device, (h) a 1D grating pixel from the proposed device, and (i) a 2D crossed‐grating pixel. Responses under unpolarized input are also plotted.

The spectral responses of the GMR PD array were measured using a monochromator scanning setup, as described in Supporting Information S1: Figure S9, with data collected at 5 nm wavelength intervals. From the measured current signals and the spectral intensity of the light source, the spectral responsivities were obtained and plotted in Figure 4f for pixels with various grating periods. The device demonstrated a maximum peak responsivity of 0.451 A/W for the pixel with a grating period of 800 nm, which is comparable to that of conventional broadband InGaAs photodetectors. The device also exhibited a low response outside the peak (approximately 0.01–0.03 A/W), confirming excellent single‐narrowband detection performance. Additional spectral response data are provided in Supporting Information S1: Figure S11. The device exhibited a maximum peak spectral external quantum efficiency (EQE) of 42.2% (Supporting Information S1: Figure S11a), indicating that more than 40% of the incident light was converted into current despite the ultrathin absorption layer. Supporting Information S1: Figure S11b, c show the noise‐measurement results and the spectral detectivity obtained from the measured current spectral density and spectral responsivity. The device exhibited a peak detectivity of 3.8 × 10^11^ Jones, which is approximately one order of magnitude lower than that of commercial InGaAs PDs [50]. Nevertheless, it demonstrates the usability of this GMR PD as a spectral sensor. Further reductions in dark current and corresponding improvements in detectivity could be achieved by improving the epitaxial growth quality of the photodetector wafer and minimizing sidewall damage introduced during mesa isolation. The full width at half maximum (FWHM) of each peak ranged from approximately 18–31 nm (Supporting Information S1: Figure S11d), which is smaller than that of conventional multispectral sensors and already comparable to that of hyperspectral sensors [1]. To evaluate the effect of grating geometry on device performance, spectral response measurements were also performed for circular‐hole grating pixels with different duty cycles, as well as for square‐hole and diamond grating pixels (Supporting Information S1: Figures S12–S14). The measurements confirmed that, as anticipated during the design stage, the circular‐hole grating pixel with a duty cycle of 0.8 exhibited the best single‐band detection performance. This is because the circular‐hole geometry provides the highest diffraction efficiency while minimizing the possibility of additional diffraction occurrence. A performance comparison between the proposed GMR PD and other reported single‐band PDs is provided in Supporting Information S1: Table S1. In addition to its high electrical performance and excellent single‐band detection performance, the proposed device offers the unique advantage of enabling multispectral pixel array fabrication through a single patterning step.

To evaluate the polarization sensitivity of the GMR PD, a linear polarizer was mounted in the measurement setup, and spectral responses were measured while rotating the polarizer in 30° increments. Figure 4g–i show the measured polarization‐dependent spectral responsivities for a pixel from the previously reported 1D grating device, a 1D grating pixel from the proposed device, and a 2D crossed‐grating pixel, respectively. For each device, the responses under unpolarized input are also plotted. The 2D grating device exhibited complete polarization independence, whereas the 1D grating devices showed peak responsivities proportional to the square of the sine or cosine of the polarization angle. Although a device exhibits single‐band characteristics, polarization dependence prevents direct acquisition of the spectral information of polarized light from a single pixel, thereby requiring multiple pixels with different orientations for polarization correction. In contrast, the proposed polarization‐independent single‐band GMR PD array enables direct acquisition of the spectral intensity at a specific wavelength from each pixel.

To verify the feasibility of the proposed device for snapshot multispectral sensing, area‐scan measurements were performed. The measurement setup is illustrated in Figure 5a. Light from a quartz tungsten halogen (QTH) lamp passed through a collimator, filters, and a SUS mask patterned with the word “light” before reaching the device. The GMR PD array was wire‐bonded to a printed circuit board (PCB) and mounted on a linear motion stage capable of movement along the *x*‐ and *y*‐axes. Each time the stage moved, the output currents from the pixels were recorded using a source meter. Three different filters were used for the measurements, and their transmittance spectra are plotted in Figure 5b. Figure 5c presents all images obtained for the three filters from six pixels with different grating periods, and Figure 5d plots the average current of each image as a function of grating period for three filters. The current for each image was determined by averaging the 10 highest values. Figure 5e shows the spectral intensities obtained from the measured current and the previously measured spectral responsivity of each pixel. The measurement results exhibit trends consistent with the transmittance spectra shown in Figure 5b. Owing to the single‐band characteristics, the spectral intensity of the light could be obtained directly from the readout of each pixel, successfully demonstrating the device's capability for real‐time snapshot multispectral sensing. However, the measured spectral intensities contain common broadband components. Although the InGaAs absorption layer was designed to be extremely thin to minimize absorption outside the resonance peak, a non‐negligible broadband absorption component remains in every pixel. To address this issue, a method for compensating for the broadband absorption component is presented in Supporting Information S1: Figure S15. Accurate multispectral sensing can be achieved by introducing a “common‐base” pixel having the same absorption spectrum as the broadband components of the other pixels and subtracting its current from that of each sensing pixel. The common‐base pixel can be designed by selecting a grating period that does not produce a resonance peak within the wavelength range of interest.

**FIGURE 5:**
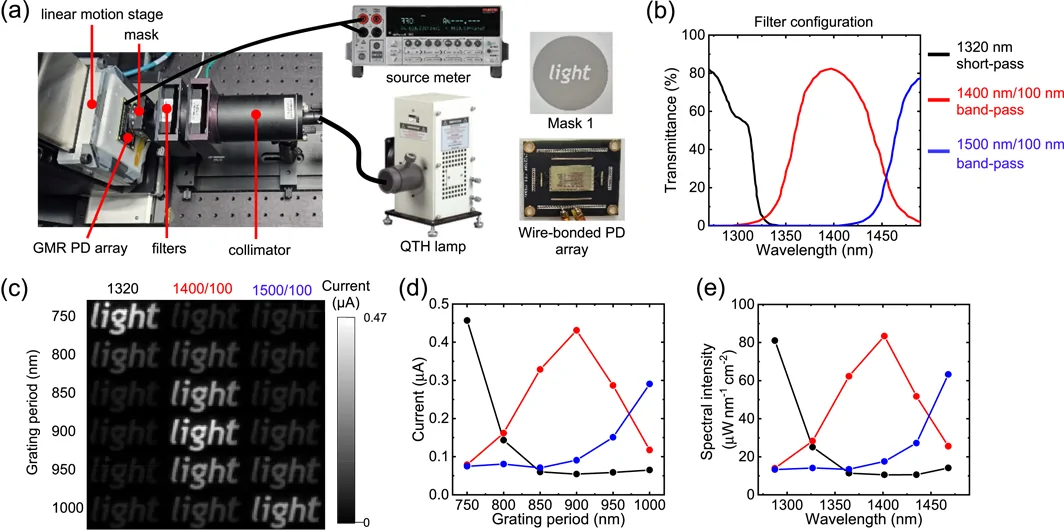
(a) Experimental setup for area‐scan measurement using a GMR PD array with a “light” mask. (b) Transmittance spectra of filters used for measurement: 1320 nm short‐pass filter, 1400 nm/100 nm band‐pass filter, and 1500 nm/100 nm band‐pass filter. (c) All images obtained for the three filters, plotted as measured currents from six pixels with different grating periods. (d) Average current of each image as a function of grating period, for three filters. The current for each image was determined by averaging the 10 highest values. (e) Obtained spectral intensities from the measured current and the spectral responsivity of each pixel.

Next, to demonstrate stable sensing performance under polarized input, measurements were performed using the mask presented in Figure 6a. Linear polarizer films were attached to the “G” and “R” regions of a “GMR” mask, with different orientations. Measurements were carried out for both 1D and 2D grating pixels with a grating period of 850 nm, and a 1200 nm long‐pass filter was mounted in the optical path. The PD array samples were attached to a rotation mount and rotated in 45° increments about an axis parallel to the incident light direction for each area‐scan measurement. The measured images are presented in Figure 6b, according to the rotation angle and grating dimension. For the 1D grating pixel, the brightness of the “G” and “R” regions with attached polarizers changes with the rotation angle of the PD. In contrast, for the 2D grating pixel, the measured image remains constant regardless of the PD angle even under polarized illumination. The average currents at the “M” and “R” regions in each image are plotted as a function of pixel angle in Figure 6c. These measurements verify the complete polarization independence of the GMR PD based on the 2D crossed grating and confirm that a GMR PD array can directly read out the spectral intensity from each pixel regardless of the polarization state of the incident light.

**FIGURE 6:**
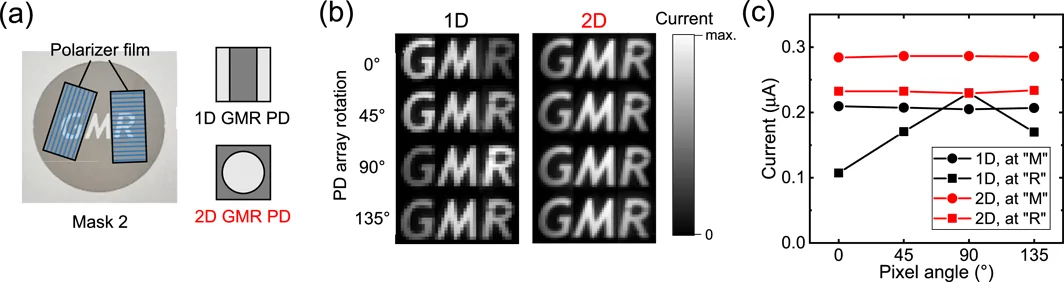
(a) Mask used for area‐scan measurement under polarized input. Linear polarizer films were attached to the “G” and “R” regions of a “GMR” mask, with different orientations. A 1/2 attenuator film (neutral density filter with optical density 0.3) was attached to the “M” region for balance. Measurements were carried out for both 1D and 2D grating pixels with a grating period of 850 nm, and their grating top view is presented. (b) The images were measured while rotating PD arrays in 45° increments about an axis parallel to the incident light. (c) The average currents at the “M” and “R” regions in each image are plotted as a function of pixel angle.

The area‐scan measurements demonstrate the feasibility of snapshot multispectral sensing using the proposed device; nevertheless, the device has two important limitations that cannot be neglected. The first is its dependence on the angle of incidence. Supporting Information S1: Figure S16 shows the simulated absorption spectra as a function of the incident angle. Owing to the intrinsic angular dispersion of GMR structures, the resonance peak splits as the incident angle increases. Therefore, an appropriate optical collimation system is required for multispectral sensing using the GMR PD array. The second limitation is the minimum pixel size. As the pixel dimensions decrease, the diffraction efficiency of the periodic structure is reduced. Moreover, because the absorber is extremely thin, the guided mode can propagate over a relatively long lateral distance, thereby requiring a sufficiently large pixel area [51]. For the device with a grating period of 850 nm, three‐dimensional FDTD simulations were performed to calculate the absorption spectrum as a function of the number of gratings contained within a pixel. The results are presented in Supporting Information S1: Figure S17. The simulated absorption spectrum begins to converge when the pixel contains approximately 20 × 20 or more grating periods. This result indicates that a device size of approximately 20 μm or larger is required to achieve maximum performance. Nevertheless, a pixel containing only about nine grating periods still exhibits a peak absorption exceeding 60% of the maximum. Incident angle dependence and pixel size limitation make the proposed device less suitable for implementing high‐resolution image sensors. However, when appropriate optical collimation is provided, the device can enable real‐time spectral analysis of incident light.

## 4 Conclusion

In this study, we presented a polarization‐independent, single‐band GMR photodetector operating in the SWIR wavelength range. By introducing a novel structure in which an InGaAs PD with an ultrathin absorption layer is bonded onto a Si grating substrate, high resonance efficiency and low off‐resonance absorption were achieved. To overcome the strong polarization dependence of conventional 1D GMR devices, we adopted a 2D crossed‐grating structure. Although 2D GMR structures inherently support a large number of optical modes, we demonstrated for the first time a single‐band 2D GMR PD exhibiting only one resonance peak over the broad wavelength range from 1100 to 1700 nm by employing new design strategies: suppressing higher‐order diffraction using an ultrathin grating layer and positioning the diffraction angle of undesired modes close to the critical angle. A GMR PD array with grating periods was fabricated, and its excellent single‐band detection performance was verified through spectral‐response measurements and area‐scan imaging. The proposed device enables fabrication of a multispectral pixel array in which each pixel has a distinct spectral band solely through grating‐period splitting. The combination of single‐band response, complete polarization independence, and a reproducible fabrication process positions this GMR PD array as a promising platform for on‐chip, real‐time multispectral sensing. Furthermore, we expect that the GMR design strategy proposed in this work will provide a foundation for advancing GMR‐based devices toward practical, high‐performance sensing applications.

## 5 Experimental Section

### 5.1 Simulation

Diffraction efficiency simulation for the 2D grating was conducted using Tidy 3D Python FDTD. Absorption simulation of the device was conducted by the rigorous coupled‐wave analysis (RCWA) method, with Rsoft CAD layout. Spectral refractive indices of InP and InGaAs were referred to Adachi, S. (1989) [52]. The output of the absorption simulation was set as the proportion of incident light absorbed at the InGaAs active layer. A wavelength resolution of the spectral absorption simulation was 1 nm. The optimization process of the device structure was conducted by repeating the absorption simulation while varying parameters.

### 5.2 Device Fabrication

The silicon‐on‐insulator (SOI) substrate was formed by chemical vapor deposition (CVD) of a 1 μm SiO_2_ box layer and a 37 nm amorphous (a‐) Si layer on a Si wafer. The grating structure was formed by photolithography patterning and a subsequent silicon dry etching process. The InGaAs PIN PD consists of layers from top to bottom as follows: *p*‐InP (23 nm)/*i*‐InP (20 nm)/*i*‐InGaAs (18 nm)/*i*‐InP (50 nm)/*n*‐InGaAs (179 nm). A 10 nm–thick *p*+ ‐InGaAs layer is located above the top *p*‐InP layer for effective ohmic contact. This epitaxial structure, grown on an InP substrate with an inverted structure, was flipped onto an SOI grating substrate by wafer bonding. Detailed epitaxial structures and the overall fabrication process are illustrated in Supporting Information S1: Figures S6 and S7, respectively. The SOI grating substrate and the InGaAs PD substrate were cleaned with acetone and methanol. After cleaning, a 10 nm–thick alumina layer was deposited by atomic layer deposition (ALD) as the bonding material on both substrates. The wafer bonding took place at room temperature after O_2_ plasma treatment, followed by heat treatment at 150°C. After wafer bonding, the InP substrate was removed by wet etching in HCl, and the etch‐stop layers were also removed. The pixel array was formed by mesa patterning and subsequent sequential wet etching using a mixed solution of hydrochloric acid and phosphoric acid for the InP layers, and a diluted mixture of phosphoric acid and hydrogen peroxide for the InGaAs layers. For the final bottom InP etching, a solution with an increased phosphoric acid ratio was used to slow the etching speed and control the etching thickness. Approximately 100 nm of the total 229 nm bottom InP layer was etched to allow bottom metal contact with *n*‐InP. After mesa formation, to passivate the mesa edges and create electrode pads, a SU‐8 layer was formed via photolithography patterning, and metal deposition was carried out to form the electrodes. The SU‐8 6000.5, an epoxy‐based permanent negative photoresist from Kayaku Advanced Materials, was used. An SU‐8 layer of about 450 nm was formed by spin coating at 3000 rpm, and after development, it was hardened by baking at 150°C. For ohmic contact, Pt was used for the top *p*+ ‐InGaAs contact and Ni was used for the bottom *n*‐InP contact. Finally, the optical window was defined by patterning and etching the *p*+ ‐InGaAs contact layer. The fabricated PD array was wire‐bonded on a PCB board for multispectral polarization sensing experiments.

### 5.3 Area‐Scan Measurements

For area‐scan measurements using the GMR PD array, a quartz tungsten halogen (QTH) lamp (6335, Newport) with a wide spectral range extending into the infrared was used as the light source. To collimate the beam and maintain good beam quality, two collimators (77,776, Newport) were used. To restrict the illumination wavelength to the target range, a 1200 nm long‐pass filter (Thorlabs), a 1310 nm short‐pass filter, a 1400–100 nm band‐pass filter, and a 1500–90 nm band‐pass filter (Newport) were employed. Two linear motion stages (MTL50, Newport) were used for area scanning. Area scanning was performed with a step size of 0.3–0.4 mm, and at each step, photocurrent signals from all pixels were measured and recorded using a source meter (Keithley 2400). To acquire multiple signals simultaneously, a DAQ6510 (Keithley) equipped with a multiplexer card was used. The masks were fabricated from a 0.5 mm–thick SUS plate with letter‐shaped apertures at the center. For measurement under polarized input, NIR linear polarizer films (25‐106, Edmund Optics) were cut and attached to the “G” and “R” regions of the mask.

## Author Contributions

**Junho Jang:** writing – original draft, investigation, conceptualization, methodology, software, data curation, formal analysis, validation, visualization, resources. **Hosang Hwang:** formal analysis, software. **SangHyeon Kim:** conceptualization, funding acquisition, writing – review and editing, project administration, supervision, resources.

## Conflicts of Interest

The authors declare no conflicts of interest.

## Supporting information


Supporting Information S1

## Data Availability

The authors declare that the data supporting the findings of this study are available within the paper and its supplementary information.
