# Supplementary material for: Polarization‐Independent Single‐Band Guided‐Mode Resonance InGaAs Photodetector Array for Snapshot Multispectral Sensing
Source: Nanophotonics. 2026 Sep 21;15(18):e70297. doi: 10.1002/nap2.70297 (PMC13672166; doi:10.1002/nap2.70297)
Supplement: Supplementary file 1 [file NAP2-15-e70297-s001.docx]

Supporting Information

Polarization-independent single-band guided-mode resonance InGaAs photodetector array for snapshot multispectral sensing

Junho Jang, Hosang Hwang, and SangHyeon Kim*

**Contents**

1. Additional absorption simulation results	2

2. Detailed structure of the proposed GMR PD	4

3. Device fabrication	5

4. Monochromator scan setup for spectral response measurement	7

5. Additional results of spectral response measurements	7

6. Performance comparison with previously reported devices	11

7. Method for compensating common broadband absorption components	13

8. Device performance according to the incident angle and the pixel size	14

**1. Additional absorption simulation results**

**Figure S1.** Spectral absorption simulation results according to the InGaAs absorption layer thickness. Absorption spectra at three thicknesses: 8 nm, 18 nm, and 30 nm are plotted in the graphs below.

Absorption

Max.

0

Period 850 nm

*t*_a_=8 nm

*t*_a_=18 nm

*t*_a_=30 nm

**Figure S2.** Spectral absorption simulation results according to the (a) PD thickness, the (b) grating height, and the (c) duty cycle.

Absorption

Max.

0

290 nm

Period 850 nm

37 nm

Period 850 nm

(a)

(b)

Period 850 nm

(c)

0.8

PD thickness 290 nm

grating height 37 nm

PD thickness 290 🡪 350 nm

grating height 37 🡪 80 nm

(a)

(b)

(c)

**Figure S3.** Simulated spectral absorption for devices with (a) originaly designed device dimension, (b) PD thickness increased to 350 nm, and (c) grating height increased to 80 nm.

**2.** **Detailed structure of the proposed GMR PD**

**Figure S4**. Unit-cell cross-section structure and detailed dimension of the propoed 2D GMR PD.

**Figure S5**. Unit-cell cross-section structure and detailed dimension of the proposed 2D GMR PD.

**3. Device fabrication**

**Figure S6.** Structure of the InGaAs PD substrate epitaxially grown on the InP wafer.

3. Wafer bonding (plasma activated)

InP substrate

optical window

*n*-InP

etch stop layers

substrate

SiO_2_ (PECVD)

Si (LPCVD)

4. Substrate removal

5. Pixel formation

alumina 10 nm

alumina 10 nm

PD substrate

1. Material deposition

2. Grating formation

(single patterning + etching)

*Λ*_1_

*Λ*_2_

*Λ*_3_

*Λ*_4_

**Figure S7.** Fabrication process of the GMR PD array.

optical window

top contact pad

bottom contact pad

*n*-InP

SU-8 passivation

**Figure S8.** Detailed schematic of a single pixel the GMR PD.

**4. Monochromator scan setup for spectral response measurement**

**Figure S9.** Schematic of the monochromator scan setup for spectral response measurement.

**Figure S10.** Images of the monochromator scan setup.

**5. Additional results of spectral response measurements**

External quantum efficiency (EQE) of the device was obtained from measured spectral responsivity, using the equation below.

$$\eta={hfR}/q$$

Where *R* is the respopnsivity, *f* is the frequancy of the light, *h* is the Plank’s constant.

Noise measurements were conducted to determine the detectivity of the device using a Keithley 4200A-SCS Parameter Analyzer. The noise floor of the measurement system is approximately 10^-15^ A Hz^-1/2^, and the dimension of the measured pixel is 168 μm×168 μm.The current spectral density was measured under dark conditions at both 0 V and -0.5 V reverse bias. The device displayed a constant current spectral density at 0 V and showed a tendency to increase inversely with frequency at -0.5 V. For the calculation of detectivity, a constant value of 2.0×10^-14^ A Hz^-1/2^ from the 0 V measurement was utilized. Spectral detectivity was obtained using the equation below.

$$D^{*}=\sqrt{A}/\mathrm{NEP}={R\sqrt{A}}/{i_{N}}$$

Where *A* is the area of the photosensitive region of the PD pixel, NEP is noise equivalent power, and *i*_N_ is the current spectral density.

Current spectral density (A Hz^-1/2^)

(a)

(b)

(c)

(d)

**Figure S11.** (a) Obtained spectral EQE of the GMR PD array, for pixels with various grating periods. (b) Measured current spectral density of the device at 0 V and -0.5 V. (c) Measured spectral responsivity and obtained spectral detectivity. (d) The FWHMs of the responsivity peaks are plotted according to the peak wavelengths.

DC 0.74

DC 0.8

DC 0.84

DC 0.89

**Figure S12.** Measured spectral responsivty of pixels with diffrent duty cycles (DCs).

**Figure S13.** SEM images of the grating substrate after grating patterning. Images of circular-hole, square-hole, and diamond gratings are presented.

1 μm

**Figure S14.** Measured spectral responsivity of the pixels with circular-hole grating, square-hole grating, and diamond grating (grating period 850 nm). For all grating patterns, area of Si and etched region (SiO_2_) are approximatelty same.

**6. Performance comparison with previously reported devices**

**Table S1.** Comparison of representative properties of the recently reported single-band PDs.

**Itallic: active devices*

[1] Q. Lin *et al*. Filterless narrowband visible photodetectors. *Nat. Photonics* **9**, 687-694 (2015)
[2] Y. Fang *et al*. Highly narrowband perovskite single-crystal photodetectors enabled by surface-charge recombination. *Nat. Photonics* **9**, 679-686 (2015)
[3] L. Shen *et al*. A filterless, visible-blind, narrow-band, and near-infrared photodetector with a gain. *Nanoscale* **8**, 12990-12997 (2016)
[4] X. Tang *et al*. Plasmon resonance enhanced colloidal HgSe quantum dot filterless narrowband photodetectors for mid-wave infrared. *J. Mater. Chem. C* **5**, 362 (2017)
[5] A. Solanki *et al*. Harnessing the interplay between photonic resonances and carrier extraction for narrowband germanium nanowire photodetectors spanning the visible to infrared. *ACS Photonics* **5**, 520-527 (2018)
[6] J. Xue *et al*. Narrowband perovskite photodetector-based image array for potential application in artificial vision. *Nano Lett.* **18**, 7628-7634 (2018)
[7] J. Wang *et al*. Organic cavity photodetectors based on nanometer-thick active layers for tunable monochromatic spectral response. *ACS Photonics* **6**, 1393-1399 (2019)
[8] J. Wang *et al*. Self-driven perovskite narrowband photodetectors with tunable spectral response. *Adv. Mater.* **33**, 2005557 (2021)
[9] P. Liu *et al*. Lead-free Cs_3_Sb_2_Br_9_ single crystals for high performance narrowband photodetector. *Adv. Optical Mater.* **8**, 2001072 (2020)
[10] Z. Liu *et al*. Narrowband near-infrared photodetector enabled by dual functional internal-filter-induced selective charge collection. *Adv. Optical Mater.* **9**, 2100288 (2021)
[11] Y. Wang *et al*. Stacked dual-wavelength near-infrared organic photodetectors. *Adv. Optical Mater.* **9**, 2001784 (2021)
[12] B. Sun *et al*. Ultra-narrowband photodetector with high responsivity enabled by integrating monolayer J-aggregate organic crystal with graphene. *Adv. Optical Mater.* **9**, 2100158 (2021)
[13] H. Wang *et al*. 2D perovskite narrowband photodetector arrays. *J. Mater.Chem. C* **9**, 11085 (2021)
[14] Z. Xu *et al*. Si/organic integrated narrowband near-infrared photodetector. *Small* **19**, 2302072 (2023)
[15] G. Kara *et al*. Metasurface cointegration with colloidal quantum dot photodiodes enabling facile spectral sensors. *IEEE International Electron Devices Meeting (IEDM) 2025*, 8-5

**Table S2.** Performance comparison with the previously reported GMR PDs.

[16] K.-W. Lai *et al*. Selecting detection wavelength of resonant cavity-enhanced photodetectors by guided-mode resonance reflectors. *Optics Express* **20**, 3572 (2012)
[17] A. Chu *et al*. Near unity absorption in nanocrystal based short wave infrared photodetectors using guided mode resonators. *ACS Photonics* **6**, 2553-2561 (2019)
[18] A. Kamboj *et al*. All-epitaxial guided-mode resonance mid-wave infrared detectors. *Appl. Phys. Lett.* **118**, 201102 (2021)
[19] A. Kamboj *et al*. Room-temperature mid-wave infrared guided-mode resonance detectors. *IEEE Photonics Technol. Lett*. **34**, 615-618 (2022)
[20] N. C. Mansfield *et al*. Room temperature mid-wave infrared guided mode resonance InAsSb photodetectors. *Appl. Phys. Lett*. **125**, 171107 (2024)
[21] N. C. Mansfield *et al*. Room-temperature mid-wave infrared guided mode resonance photodetectors for focal plane arrays. *IEEE J. Sel. Top. Quantum Electron*. **32**, 3800509 (2026)
[22] J. Jang *et al*. Guided-mode resonance polarization-sensitive narrowband InGaAs photodetector. *Laser Photonics Rev.* **19**, 2401253 (2025)

**7. Method for compensating common broadband absorption components**

**Figure S15.** Simulated absorption spectra of device with grating period 850 nm and 350 nm. The “common-base“ pixel, which has the same absorption spectrum as the broadband components of the other pixels, can be designed by selecting a grating period that does not produce a resonance peak within the wavelength range of interest.

**8. Device performance according to the incident angle and the pixel size**

Absorption

Max.

0

Period 850 nm

*θ*

light incidence

unit cell structure

**Figure S16.** Simulated absorption spectra according to the incident angle.

simulation boundary

plane source

monitor 1

monitor 2

Si grating layer

InP-InGaAs PD

SiO_2_

Si grating layer (13 x 13 gratings)

(b)

(c)

(d)

(a)

**Figure S17.** (a) Ansys Lumerical FDTD simulation window. (b) Image of the Si grating layer contains 13 x 13 gratings. (c) Simulated absorption spectra according to the number of gratings in a pixel. (d) Simulated peak absorption according to the number of gratings.
